# Validation and application of a standardized quantitative PCR assay for the assessment of antimicrobial resistance genes in surface water

**DOI:** 10.1038/s41598-026-35635-x

**Published:** 2026-03-02

**Authors:** Laura C. Scott, Christina A. Ahlstrom, Hanna Woksepp, Jonas Bonnedahl, Cherie M. McKeeman, Mark E. Miller, Dave Schirokauer, Andrew M. Ramey

**Affiliations:** 1 U.S. Geological Survey, Alaska Science Center, Anchorage, AK USA; 2 Department of Clinical Microbiology, Region Kalmar County, 391 85 Kalmar, Sweden; 3 Department of Biomedical and Clinical Sciences, Linköping University, Linköping, Sweden; 4 Department of Infectious Diseases, Region Kalmar County, Kalmar, Sweden; 5 National Park Service, Wrangell-St. Elias National Park and Preserve, Copper Center, AK USA; 6 National Park Service, Denali National Park and Preserve, Denali Park, AK USA

**Keywords:** Antimicrobial resistance, One health, Quantitative PCR, Microbial source tracking, Water quality, Biological techniques, Biotechnology, Environmental sciences, Microbiology, Molecular biology

## Abstract

Antimicrobial resistance can be an indicator of anthropogenic contamination in surface waters and is a potential public health threat. Methodological standardization for characterization of antimicrobial resistance in the environment is lacking. Quantitative PCR (qPCR) is used for rapid assessment of antibiotic resistance genes (ARGs) from environmental sources, including surface water. Here we describe the validation and application of a qPCR assay for 47 bacterial gene targets intended for surface water samples. The qPCR assay displayed excellent sensitivity (97.66%) and specificity (98.71%) for detecting ARGs when compared to whole genome sequencing of bacterial isolates. The qPCR assay was able to detect up to 6/8 (75.0%) of ARGs spiked into sterile water at varying concentrations and four sample ultrafiltration volumes. Nineteen different ARGs were detected across six samples sites at three national parks in Alaska using ultrafiltered surface water samples. The number of unique ARGs detected was higher at sites within parks with greater visitation. The relative abundance of ARGs/16S from Exit Creek in Kenai Fjords National Park, downstream from a visitor center was greater than all other sampled sites. We have demonstrated a robust qPCR assay for monitoring ARGs in surface waters, including those that are minimally human impacted.

**Supplementary Information:**

The online version contains supplementary material available at 10.1038/s41598-026-35635-x.

## Introduction

Assessment of antimicrobial resistance (AMR) in surface waters is a common approach used for One Health focused investigations of AMR. However, methodological standardization remains a hurdle to cohesive characterizations of environmental AMR^1–5^. The Environmental Working Group of the National Antimicrobial Resistance Monitoring System (NARMS) in the United States recently published guidelines and recommendations for monitoring AMR in freshwaters including study design strategies, selection of AMR indicators, and analytical methods^1^. Concurrent utilization of methods assessing phenotypic AMR and genomic methods is a common thread among calls for standardization^1,2,6^.

Human and agricultural wastewater effluents are important sources of antibiotic resistance genes (ARGs) in surface waters^7–10^. Similarly, land-use is correlated with differences in ARG profiles in surface waters^11–13^. Measuring indicators of AMR along human impact gradients in individual watersheds has elucidated environmental transmission mechanisms^7,14–17^, but baseline AMR characterizations are critical for downstream interpretations^11,18–20^. National parks are often prime examples of areas exhibiting gradients of human impact given that many parks have areas of extreme popularity and high visitation alongside inaccessible, sparsely visited areas. Previous research has demonstrated that visitation impacts surface water quality in national parks^21–24^, and the occurrence of AMR can be used as a metric of anthropogenic contamination in park waters^25–27^. Characterization of AMR may help public land managers balance assessment and management of natural resource contamination alongside monitoring for health hazards. Quantitative PCR (qPCR) has been used extensively for the detection of ARGs both clinically and in the environment, including the development of published and commercially manufactured qPCR assays for the detection of ARGs^28–32^. However, commercially available assays are clinically focused and do not include targets for research questions of both environmental and clinical significance^28^. While many published studies exist for the assessment of ARGs in the environment, no standard targets or primer sets exist to our knowledge.

Dead-end hollow-fiber ultrafiltration has potential for use in standardized assessment of AMR among surface waters. Dead-end hollow-fiber ultrafiltration is an established method for the collection of large volume samples from water for a wide variety of microbial analyses, utilizing hemodialysis filters (ultrafilters)^33–42^. An inherent benefit of ultrafiltration is the ability to sample large volumes of water in search of rare targets. Previous studies of groundwater, partially treated drinking water, and tap water were able to sample up to 1,517 L, 500 L, and 100 L respectively, with ultrafiltration to assess the presence of microbial targets^11,40,43^. Bacterial recovery has been estimated to be between 75%^41^ and > 95%^37^ when using ultrafiltration. Hollow-fiber ultrafiltration can also withstand high particulate matter or turbidity that often occurs with surface water sampling^44,45^.

In this study, we build upon the NARMS recommendations by investigating and optimizing established sampling and quantitative PCR (qPCR) methods for the assessment of AMR indicators in surface waters, including those lacking obvious anthropogenic inputs. Our goal was to develop an assay able to provide inference on types of AMR present in surface waters and their origin (e.g., natural background vs. anthropogenic contamination). Specifically, we developed a single plate qPCR assay for the detection of antibiotic resistance genes (ARGs) of clinical and environmental significance and microbial source tracking markers. Herein we describe this assay and its validation. We assessed the recovery of bacterial gene targets using dead-end hollow-fiber ultrafiltration from low biomass spiked samples and applied these methods to detect AMR in surface water at three national parks in Alaska, USA.

## Materials and methods

### Development of the AMR qPCR assay

Primer sets for 44 antimicrobial resistance gene targets were selected from previous studies, the Resistomap platform (Resistomap, Oy, Helsinki, Finland), or developed in this study (Supplementary Table 1): aminoglycoside resistance genes (*aac3-IV*,* aac(6’)-IIc*,* aacA43*,* APH*,* rmtB*), betalactam resistance genes (*bla*_*CMY*_, *bla*_*GES*_, *bla*_*IMP*_, *bla*_*KPC*_, *bla*_*NDM*_, *bla*_*OX*_, *bla*_*OXY*_, *bla*_*SHV*_, *bla*_*TEM*_, *bla*_*VIM*_, *bla*_*CTX−M*_), class one integrons (*dfrA1*,* int1*), multidrug efflux pumps (*qacEdelta*,* mepR*,* mexF*), glycopeptide resistance genes (*vanA*,* vanD*), macrolide resistance genes (*ereA*,* ermB*,* ermD*,* ermF*), mobile genetic elements (*IS613*), phenicol resistance genes (*catII*,* catP*), polymyxin resistance genes (*mcr-1*,* mcr-2*,* mcr-3*), quinolone resistance genes (*oqxA*,* qnrB*,* qnrS*), sulfonamide resistance genes (*sul1*,* sul3*), and tetracycline resistance genes (*tetA*,* tetD*,* tetM*,* tetL*,* tetX*). Microbial source trackers were also selected for humans (*HF183*), birds (*GFD*), and ruminants (*Rum2Bac*) and the universal bacteria marker (16 S rRNA^46^). Primer sets for *bla*_*GES*_, *bla*_*IMP*_, *bla*_*KPC*_, *bla*_*OXY*_, *bla*_*VIM*_, *bla*_*CTX−M*_, *dfrA1*, *ereA*, *ermB*, *ermF*, *mcr-1*, *mcr-3*, *qacEdelta*, *qnrB*, *sul3*, and *tetX* were developed in this study. Primer sets developed in this study were generated by identifying each gene target nucleotide sequence in the National Center for Biotechnology Information (NCBI) Bacterial Antimicrobial Resistance Reference Gene Database^47^. The NCBI Primer Blast tool was used to find suitable qPCR primer sets within the gene targets sequences, limiting amplicons to a base pair length of 100–300 bp. All ARG qPCR templates were queried through the Comprehensive Antibiotic Resistance Database (CARD)^48^ to confirm 100% identity to the anticipated gene target. The resulting primer sets and templates are reported in Supplementary Table (1) Targets with matches to multiple gene variants were recorded and are reported in Supplementary Table (2) Amplicon sequences for 16 S, microbial source trackers, mobile genetic elements, and class one integrons were confirmed with NCBI BLAST^47^.

Standard curves were generated across 8 logs (10^7^ gene copies/µL – 10^0^ gene copies/µL) of 10-fold dilutions of synthetic genomic DNA templates (gBlocks) (Integrated DNA Technologies) in triplicate. Each qPCR reaction was done in 20-µL volumes with the following inclusions: 10 µL of 2x PowerUp SYBR Mastermix (Applied Biosystems), 6 µL of nanopure water, 2 µL of template DNA, and 1 µL of each forward and reverse primer. Final primer concentrations were 0.5 µM and are listed for each target in Supplementary Table 1. Thermal cycling conditions for the qPCR reaction were as follows: uracil-DNA glycosylase deactivation at 50℃ for 2 min, polymerase activation at 95℃ for 2 min, 40 cycles of 95℃ for 15 s followed by 60℃ for 30 s, and a melt curve analysis of 95℃ for 1 min, 60℃ for 5 min, and gradual increase of temperature from 60℃ to 95℃ at 0.1℃/second. For every sample, every target was run in duplicate, and every plate included duplicate non-template control wells. The limit of detection for each target was designated as the lowest concentration that was detected in every standard curve replicate.

### Comparison of ARG detection using qPCR and whole genome sequencing of bacterial isolates

To quantify the sensitivity and specificity of the qPCR assay for the detection of ARGs, detection of the 44 AMR targets were compared using DNA from 41 *Escherichia coli* and *Klebsiella pneumoniae* isolates initially selected for the comparison of a commercially available qPCR assay to whole genome sequencing (WGS) in prior research^28^. DNA from each isolate was run in duplicate with the previously described AMR assay on a BioRad Laboratories (Hercules, California) CFX-96 RT-PCR Detection System. Samples were considered positive for an ARG target when both replicates returned a Ct value greater than the limit of detection for that target. Target detection through whole genome sequencing was done using ResFinder^49^ as previously described^28^. Sensitivity, specificity, and Cohen’s Kappa were calculated for the qPCR assay compared to WGS in SAS 9.4 software with the “proc freq” command^50^.

### Laboratory assessment of ultrafiltration and qPCR for detection of ARGs from water samples

An experiment utilizing sterile water spiked with known bacteria at three concentrations was done to better estimate the variation and detection limits of ultrafiltration sampling for qPCR detection of rare target ARGs in water samples. Six bacteria were chosen from the American Type Culture Catalogue (ATCC, Manassas, Virginia) for containing at least one of the AMR targets included in the qPCR assay. Eight ARGs were represented across the six bacteria: *bla*_*TEM*_, *dfrA1*, *ermB*, *ermD*, *intI1*, *mepR*, *qacEdelta*, and *sul1* (Supplementary Table 3). Bacteria were grown to log phase cultures as described by the manufacturer. Individual cultures were mixed thoroughly, and 1 mL was removed from each for DNA extraction. DNA was extracted using the DNeasy PowerWater kit (Qiagen, Hilden, Germany) following manufacturer instructions on a Qiacube system. DNA from each bacterium was assessed for ARGs using the qPCR assay described previously. Bacterial concentration for each culture was estimated from 16 S rRNA copies detected with qPCR and the known number of 16 S rRNA copies held per genome for each species.

Nanopure water was measured into 50 L, 25 L, 10 L, and 1 L volumes. Bacterial cultures were prepared to create mixtures of roughly 10,000 cells per liter, 1,000 cells per liter, and 100 cells per liter of each bacterium within each volume of water. At each volume, bacterial spikes were conducted for each concentration in triplicate (*n* = 36). Bacterial spikes were prepared immediately prior to ultrafiltration to avoid significant concentration changes between inoculation and filtration. Each spiked water replicate was filtered across an ELISIO 25 H ultrafilter with a peristaltic pump (Geotech Environmental Equipment, Denver, Colorado) set at approximately 600 rpm. Filters were immediately backflushed with a peristaltic pump set at approximately 600 rpm with 500 mL of 0.5% Tween 80/ 0.01% Sodium polyphosphate/0.001% Antifoam Y-30^42^. A 250-mL volume of backflushed eluate was secondarily concentrated across 0.2 μm polycarbonate membrane filters (Millipore Sigma, Burlington, Massachusetts) in duplicate. DNA was extracted from membrane filters using the DNeasy PowerWater extraction kit following manufacturer’s protocols on a Qiacube system (Qiagen). DNA extracts were assessed for ARGs using the qPCR AMR assay on a QuantStudio 12 K Flex (Applied Biosystems, Waltham, Massachusetts). A gene amplification above the threshold in both qPCR replicate wells and exhibiting the correct melt curve temperature was designated a detection of high confidence. When a sample was detected in only one qPCR replicate well and had the correct melt curve temperature, it was reported as a presumptive positive.

### Assessment of AMR indicators in surface water from three National parks

**Fig. 1:**
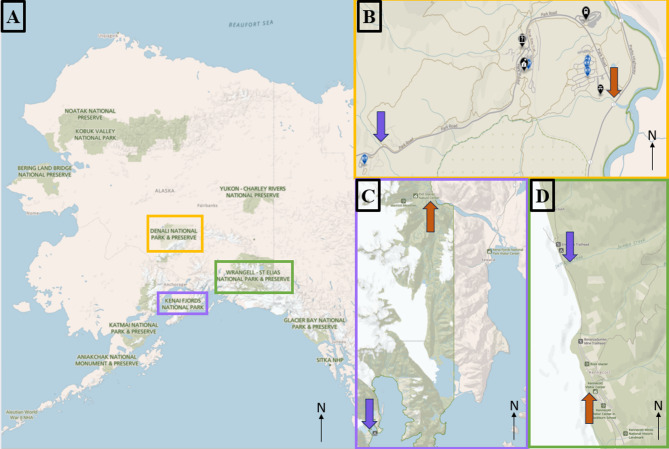
**A** National Park Service units in Alaska. Parks sampled in this study are boxed in yellow (Denali National Park), purple (Kenai Fjords National Park), and green (Wrangell-St. Elias National Park). **B**–**D** Sampling sites within Denali National Park at Rock Creek (purple) and Riley Creek (orange) (**B**), Kenai Fjords National Park at Surprise Glacier (purple) and Exit Creek (orange) (**C**), and Wrangell-St. Elias National Park at Jumbo Creek (purple) and National Creek (orange) (**D**). Within each park, sites of lower perceived human impact are approximately marked with a purple arrow, and sites of higher human impact approximately marked with an orange arrow. The base layer map was produced by the National Park Service and can be accessed at https://www.nps.gov/anch/planyourvisit/maps.htm.

We applied the ultrafiltration and qPCR assay to an investigation of AMR in three national parks in Alaska. We sampled two streams from three national parks of varying visitation in Alaska: Kenai Fjords National Park, Denali National Park, and Wrangell St. Elias National Park. In 2023 the number of recorded visitors in each park was 498,722 in Denali National Park, 387,525 in Kenai Fjords National Park, and 78,305 in Wrangell-St. Elias National Park^51^. Two sampling sites were selected in each park, one of likely higher human impact and one of likely lower human impact (Fig. 1). Site proximity to roadways, visitor centers, and trailheads were considered in estimating human impact for sampling sites. In Kenai Fjords National Park, we sampled at (1) Exit Creek (higher impact) downstream of a visitor center, trailhead, and campground and (2) a tidally influenced creek (lower impact) below Surprise Glacier located along the western margin of a remote fjord southwest of Seward. In Denali National Park, samples were collected from (1) Riley Creek (higher impact), downstream of a large front-country campground and (2) Rock Creek (lower impact) which is downstream of several in-park residences including staff housing and private residences. In Wrangell-St. Elias National Park, we sampled (1) National Creek (higher impact), downstream of an historic copper mill and various National Park Service-managed facilities and (2) Jumbo Creek (lower impact), downstream of an historic copper mine along a hiking trail. At each site, triplicate 20-L samples and duplicate 50-L samples were filtered on-site using a Geopump Series II peristaltic pump (Geotech Environmental Equipment, Denver, Colorado). Filters were refrigerated until they were returned to the laboratory and backflushed within 72 h of sampling. Backflush was divided into 250-mL replicates and secondarily concentrated across 0.2 μm polycarbonate membrane filters. One filter was used for culture and phenotypical analysis of AMR bacteria and the other was DNA extracted and examined with qPCR for ARGs as previously described on a QuantStudio 12 K Flex.

Phenotypic characterization of the samples was carried out by inoculating half of a filter into 5 mL of buffered peptone water (BPW) (ReadyTube^®^, Merck KGaA, Darmstadt, Germany) and the other half into 5 mL of Rappaport-Vassiliadis soya peptone (RSV) broth (Oxoid, Thermo Fisher Diagnostics, Waltham, Massachusetts). The samples were incubated at 36 °C (± 1 °C) at 70 rpm for 18–24 h. After incubation, 10 µL of the BPW inoculum was spread onto non-selective media (Orientation, CHROMagar™, EMMlife Science, Stockholm, Sweden) and selective media designed for the isolation of bacteria such as extended-spectrum beta-lactamase (ESBL) producers, carbapenemase-resistant *Enterobacteriaceae* (CRE), methicillin-resistant *Staphylococcus aureus* (MRSA), vancomycin-resistant *enterococci* (VRE), and *Salmonella* spp.The selective agar plates used included ESBL, mSuperCarba™, and MRSA (CHROMagar™, EMMlife Science), VRE (chromID^®^, bioMérieux, Marcy-l’Étoile, France), Xylose Lysine Deoxycholate (XLD) (E&O Laboratories Ltd., Burnhouse, Bonnybridge, Falkirk, Scotland), and Deoxycholate Citrate Lactose (DC) (BioRad Laboratories). Additionally, BPW inoculum was spread onto one Mueller Hinton (MH) Orientation plate (CHROMagar™, EMMlife Science) with antibiotic discs containing ciprofloxacin (5 µg), erythromycin (15 µg), and tobramycin (10 µg) (all from Oxoid, Thermo Fisher Diagnostics). XLD and DC plates were also inoculated with samples enriched in RSV broth. All plates were incubated at 36 °C (± 1 °C) for 18–24 h. Bacterial colonies with distinct morphologies were isolated from the non-selective media, and species identification was performed using MALDI-TOF MS (Bruker Corporation, Billerica, Massachusetts). Putative resistant bacteria were isolated from the respective selective media and from zones of bacterial growth around the antibiotic discs. Species identification was again performed using MALDI-TOF MS. The XLD and DC media were inspected for potential growth of *Salmonella* spp. Antimicrobial susceptibility testing (AST) and breakpoint interpretation were conducted according to EUCAST guidelines^52^.

Bacterial community composition was assessed for each sample by sequencing the V3 and V4 regions of 16 S rRNA^53^ using remaining extracted DNA from prior analyses (Supplementary Table 4). In the case of Exit Creek from Kenai Fjords National Park, DNA from only one replicate remained for metagenomic analysis. Libraries were prepared following the Illumina 16 S Metagenomic Sequencing Library Preparation Guide and sequenced with 600 cycles on a NextSeq 1000 in duplicate (Illumina, Inc., San Diego, California)^54^. Raw reads were characterized using Kraken2 (v2.1.3)^55^ and the RefSeq complete bacterial genomes reference library, downloaded on August 9, 2024^55^. Reads with a quality score of less than 20 were excluded. Sample output reports were combined using kraken-biom^57^ and visualized as a heatmap of relative read abundance and principal component analysis plot using the argument amp_heatmap and amp_ordinate, respectively, using the distance measure bray and default settings in the package ampviz2^58^ in R version 4.2.0^59^.

### Statistical analysis

To gain inference on the relative abundance of ARGs between sites, one-way ANOVA, using Tukey’s multiple comparisons test, was conducted using all detected ARG/16S rRNA ratios from each national park sampling site. Statistical significance was set at an alpha equal to 0.05. For this analysis, ARG and 16 S concentrations were averaged across high confidence detections from all sample volumes and replicates of the same site. Statistical analyses were conducted in GraphPad Prism version 10.2.0 (Boston, Massachusetts).

## Results

### Comparison of ARG detection using qPCR to whole genome sequencing of bacterial isolates

DNA from 41 bacterial isolates was assessed for 43 ARGs using qPCR and WGS. When comparing the qPCR assay to WGS, the two methods had 98.58% (1,738/1,763) agreement, and Cohen’s Kappa agreement was 0.9355 (95% confidence intervals [CI]: 0.9104–0.9605). The estimated sensitivity and specificity of the qPCR were measured to be 97.66% ([CI: 95.64%–99.69%) and 98.71% ([CI]: 98.15%–99.27%) respectively. The 20 noncongruent positive qPCR detections were distributed across eight targets: *aacA43* (*n* = 4), *bla*_*SHV*_ (*n* = 1), *bla*_*TEM*_ (*n* = 2), *ereA* (*n* = 2), *ermB* (*n* = 1), *int1* (*n* = 2), *qacEdelta* (*n* = 7), and *tetD* (*n* = 1), while the five noncongruent non-detections occurred for four gene targets: *bla*_*TEM*_ (*n* = 1), *catII* (*n* = 1), *qnrB* (*n* = 2), and *tetA* (*n* = 1)^60^. Detection probability was calculated for the entire assay by dividing the number of observed congruent qPCR and WGS detections by the total number of WGS positive detections and was 92.59% (250/270).

### Laboratory assessment of ultrafiltration and qPCR for detection of ARGs from water samples

**Fig. 2:**
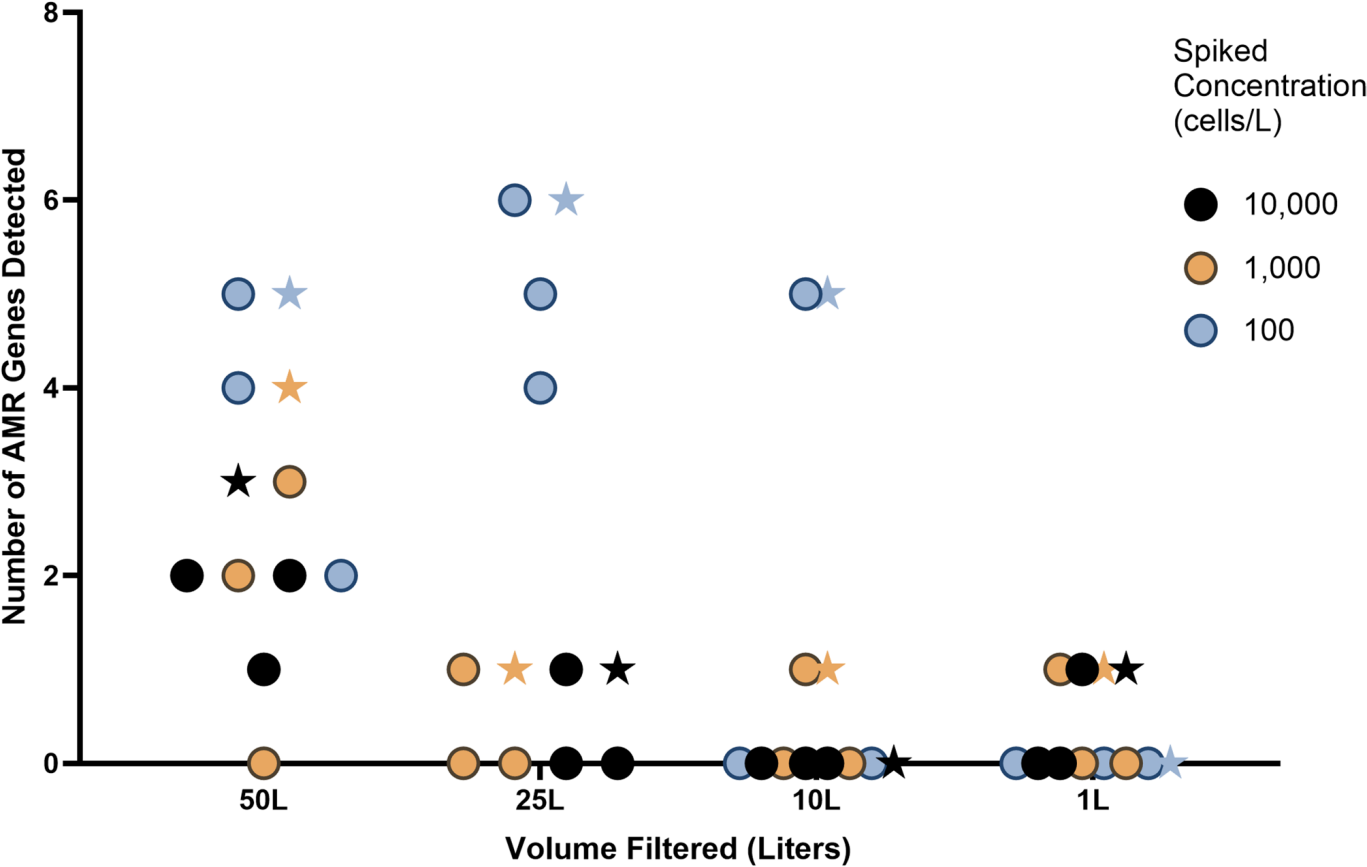
Number of antimicrobial resistance gene targets detected by qPCR with high confidence from four volumes of laboratory water spiked with bacteria at three concentrations. Dots represent individual replicate samples. Sample points shaped as a star indicate the outcome of pooled analysis.

qPCR of extracted DNA from each bacterial culture confirmed the presence of all ARGs reported to be present within each bacterium, and no other ARGs were detected. Sterilized water was spiked with bacteria and analyzed by the previously described qPCR method for the detection of ARGs. The 16 S target was detected with high confidence in every replicate at all volumes and spike in concentrations. Results from triplicate samples of the same volume and spike concentration were pooled and analyzed as one to mimic intended field strategies. In the 50-L triplicate pool, five (62.5%), four (50.0%), and three (37.5%) of eight spiked ARGs were detected with high confidence from the 10,000 cells per liter, 1,000 cells per liter, and 100 cells per liter spikes, respectively (Fig. 2). When including presumptive detections, four (50.0%) and five (62.5%) of ARGs were detected from the 10,000 cells per liter and 1,000 cells per liter spiked 50 L pooled samples, respectively (Supplementary Fig. 1). At least one ARG was detected with high confidence in every spiked concentration at 50 L and 25 L. The most high-confidence detections of ARGs in the 50-L, 25-L, and 10-L samples were at the 100 cells per liter spiked concentration (Fig. 2).

ARG detection was also analyzed per filter replicate. At least one ARG was detected with high confidence or presumptively in every 50-L replicate regardless of spike concentration (Supplementary Fig. 1). Out of eight expected ARGs, the number of high-confidence ARG detections at the highest concentration in 50-L replicates were two (25.0%), one (12.5%), and two (25.0%). Conversely, ARGs were only detected with high confidence in two one-liter replicates, at 1,000 cells/L and 10,000 cells/L, and one ARG (12.5%) was detected in each. The greatest number of ARGs detected with high confidence was six (75.0%) in a 25-L replicate at the 100 cells per liter concentration (Fig. 2). The number of replicates with zero detections ranged from one (in the 50-L sample) to seven (in the 10-L and 1-L samples) (Supplementary Fig. 1).

**Fig. 3:**
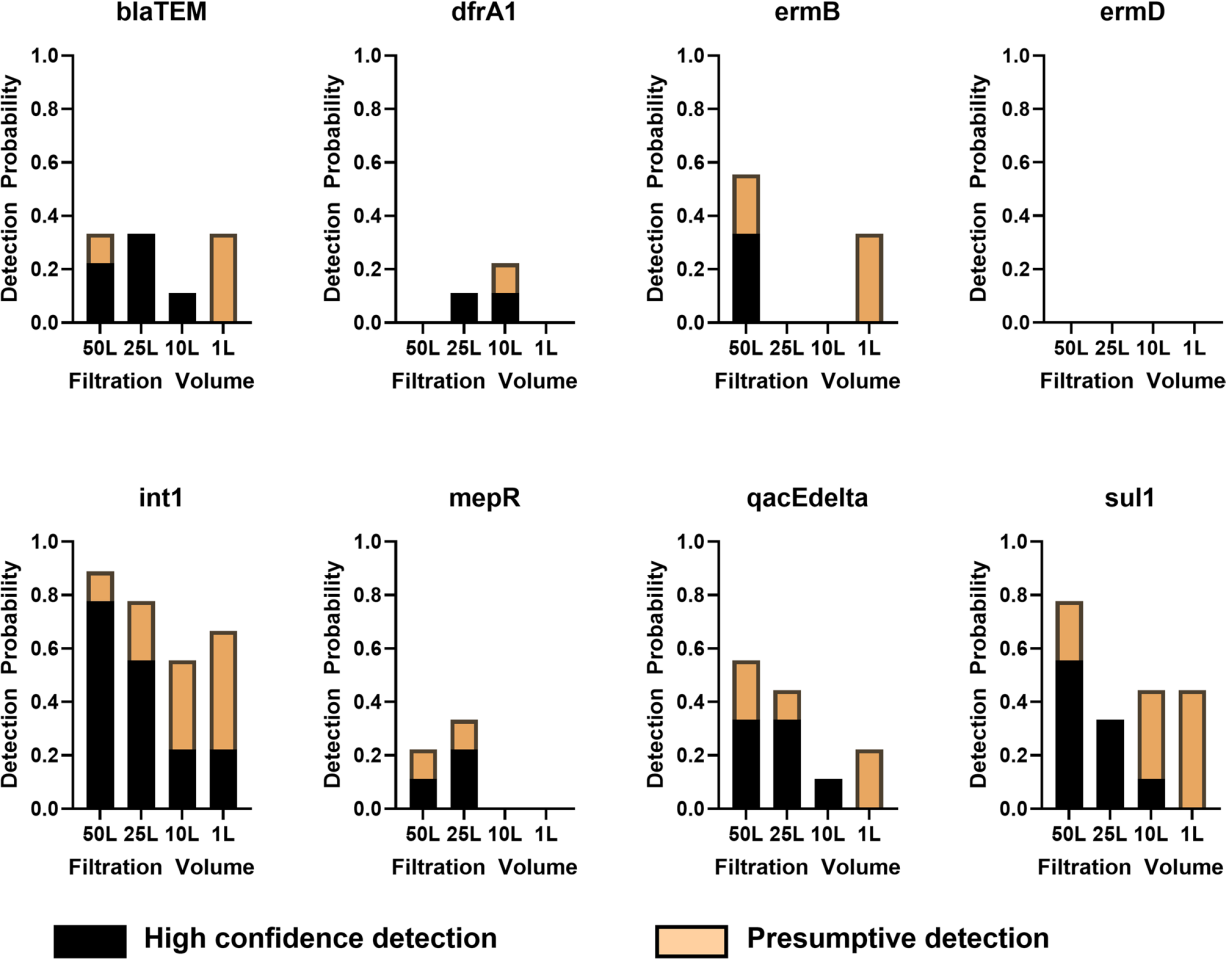
Detection probabilities of gene targets spiked into 50-, 25-, 10-, and 1-liter sterile water samples, ultrafiltered, and assessed with qPCR.

Detection probabilities were calculated for each ARG by dividing the number of instances the gene was detected with high confidence or presumptively by the total number of times it was expected. Probability of detection varied among ARGs (Fig. 3). The class one integron gene, *int1*, had the highest detection probabilities among all filtration volumes, while *ermD* was never recovered. ARGs that were held by more than two of the spiked species (e.g., *int1*, *qacEdelta*, *sul1*) generally had higher detection probabilities (Supplementary Fig. 2). 100% high-confidence detection probabilities were calculated for 25 L and 100 cells per liter for *bla*_*TEM*_, *sul1*, *int1*, and *qacEdelta* (Supplementary Fig. 3). When including presumptive and high confidence detections, at least one combination of sample volume and spike concentration had a greater than 50.0% detection probability for *sul1*, *bla*_*TEM*_, *int1*, *ermB*, *qacEdelta*, and *mepR* gene targets (Supplementary Fig. 3).

We assessed the relative ARG quantities determined from the ultrafiltration and qPCR process by comparing the ratio of measured ARG concentrations (copies per liter) to 16 S rRNA concentrations (copies per liter) from each filter yielding a high confidence detection to the expected ARG/16S rRNA ratio based upon the spike-in concentration derived from known 16 S rRNA copy numbers held by each species. The average difference between measured and expected ARG/16S rRNA ratios for the seven targets with high confidence detections was 0.020 and ranged from − 0.091 to 0.066 (Supplementary Fig. 4). The measured ratios were higher than the expected ratios for *bla*_*TEM*_ and *dfrA1*.

### qPCR, phenotypic screening, and metagenomics to characterize ARGs and bacterial diversity in three National parks

**Fig. 4:**
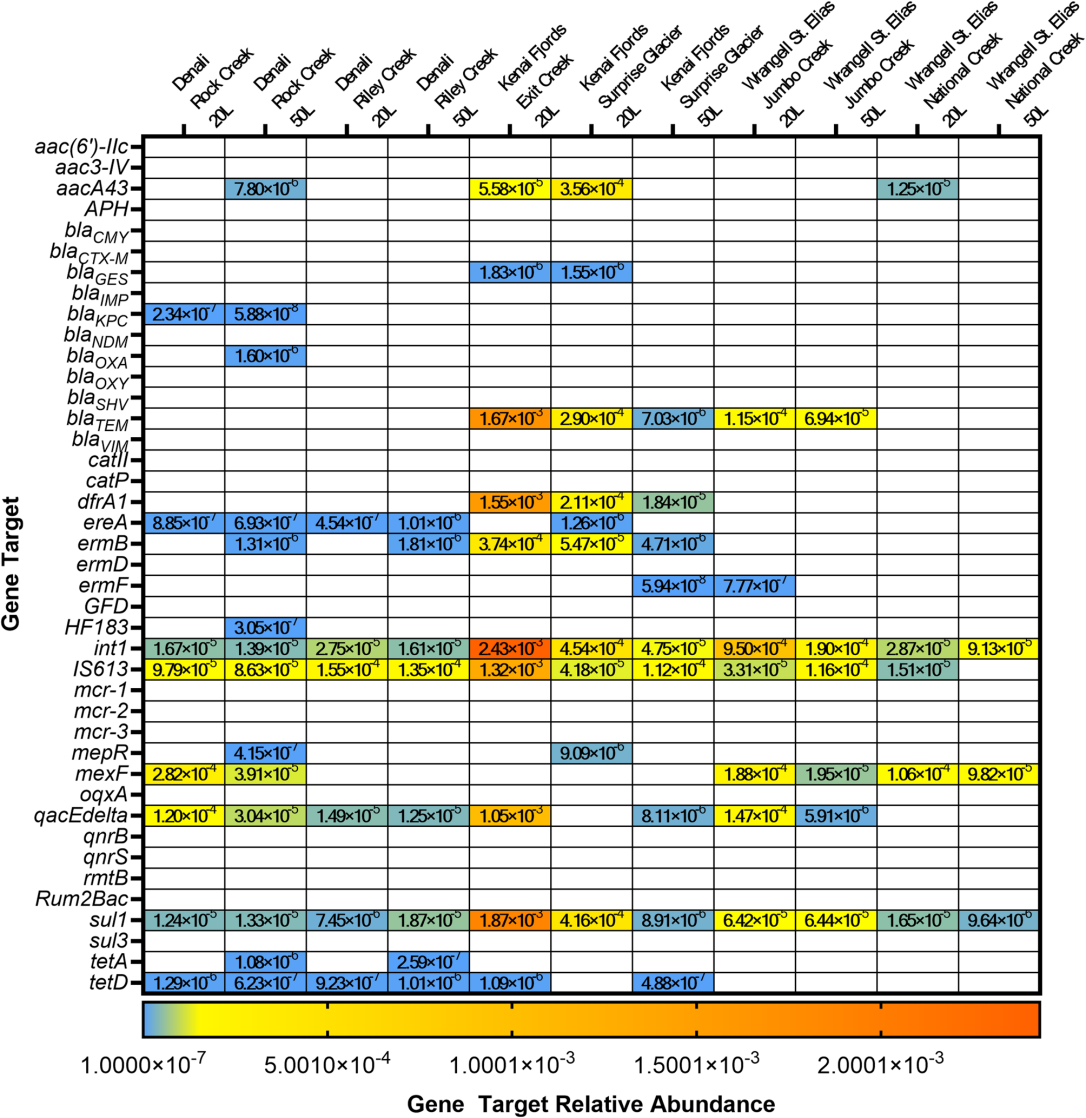
High-confidence detections and relative abundances of antimicrobial resistance genes from 20-L and 50-L ultrafiltered surface water replicates from six sites at three national parks in Alaska.

Given the demonstrated variability in gene target detection between replicates in the spikes, we chose to take field replicates for two sampling volumes at each site. Water was filtered in 20-L triplicate and 50-L duplicate volumes at two sites each in three national parks in Alaska with one exception. No 50-L replicates could be taken at Exit Creek (KEFJ) due to filter clogging, and only the 20-L triplicates were achieved. Filters from secondary concentration were either subjected to bacterial culture for phenotypic antibiotic susceptibility testing or DNA extraction for qPCR. Nineteen unique gene targets were detected with high confidence, and an additional two targets were presumptively detected (Fig. 4). Target bacteria were isolated from every site except Exit Creek at Kenai Fjords National Park (Supplementary Table 4); however, only a single isolated demonstrated phenotypic antimicrobial resistance. An *E. coli* isolate from Riley Creek at Denali National Park demonstrated phenotypic resistance to ampicillin.

Detection outcomes were compared between 20-L and 50-L replicates from the same site by assessing whether a gene was detected with high confidence in any of the replicates of the same volume. Of the 240 comparisons made, there were 18 (7.5%) instances of incongruent detection. Of these disagreements, 61.1% (*n* = 11) occurred due to a gene target being detected in a 50-L sample but not being detected in a 20-L sample (Fig. 4). Replicability of detection was compared among filter replicates from the same site of the same sample volume. If a target was detected with high confidence in any replicate of any sample volume at a site, replicability within same-volume filter replicates was assessed (Supplementary Fig. 5A). Congruent high confidence detections among replicates occurred more often in 50-L samples (*n* = 18) than 20-L samples (*n* = 9). The percent of detections classified as high-confidence detections were also compared between 20-L and 50-L replicates from the same sites (Supplementary Fig. 5B). The mean proportion of high-confidence detections in 20-L and 50-L samples were 0.8281 and 0.0.8724, respectively. Twenty-one total presumptive detections were observed from 20-L samples and seven presumptive detections were observed from 50-L samples. The standard deviations of relative gene target abundance were calculated by dividing measured gene target copies/L by measured 16 S rRNA copies/L and were significantly greater among 20-L replicates than 50-L replicates when compared with a t-test (*p* = 0.0226) (Supplementary Fig. 5C).

The greatest number of unique gene detections was at Rock Creek in Denali National Park (high confidence *n* = 15), and the fewest unique genes detected was at National Creek in Wrangell St. Elias National Park (high confidence *n* = 6) (Fig. 4). For Kenai Fjords National Park, the creek below Surprise Glacier, had more unique target detections (high confidence *n* = 14) than Exit Creek (high confidence *n* = 11); however, the number of detections were equivalent between sites when controlling for sample volume filtered. The number of unique, high-confidence, or presumptive gene target detections from all volumes and replicates in Kenai Fjords National Park, Denali National Park, and Wrangell-St. Elias National Park were 14, 16, and 11, respectively (Supplementary Fig. 6).

The only gene target detected with high confidence in every replicate at every site was 16 S rRNA. However, the targets *int1*, *IS613*, and *sul1* were detected with high confidence in at least one replicate from every site (Fig. 4). Tetracycline resistance genes were detected at multiple sites. *TetA* was detected only in Denali National Park at both Rock Creek and Riley Creek. The tetracycline resistance gene *tetD* was detected with high confidence in multiple replicates at both Denali sites, in a single replicate at both Kenai Fjords sites, and in no replicates at either Wrangell-St. Elias site (Supplementary Fig. 6). Only two detections of microbial source trackers occurred. The human marker (*HF183*) was detected with high confidence, and the avian marker (*GFD*) was presumptively detected at Rock Creek. Of the betalactamase resistance genes, *bla*_*TEM*_ was the most common, with two high confidence detection replicates at Exit Creek and the creek below Surprise Glacier in Kenai Fjords National Park and Jumbo Creek at Wrangell-St. Elias National Park. The carbapenemase resistance gene, *bla*_*KPC*_, was detected with high confidence in two replicates at Rock Creek in Denali National Park and presumptively detected in one replicate in the creek below Surprise Glacier in Kenai Fjords National Park (Supplementary Fig. 6). Two other betalactamase resistance genes, *bla*_*OXA*_ and *bla*_*CTX−M*_, were detected presumptively at Riley Creek in Denali National Park and *bla*_*OXA*_ was detected with high confidence at Rock Creek in Denali National Park.

**Fig. 5:**
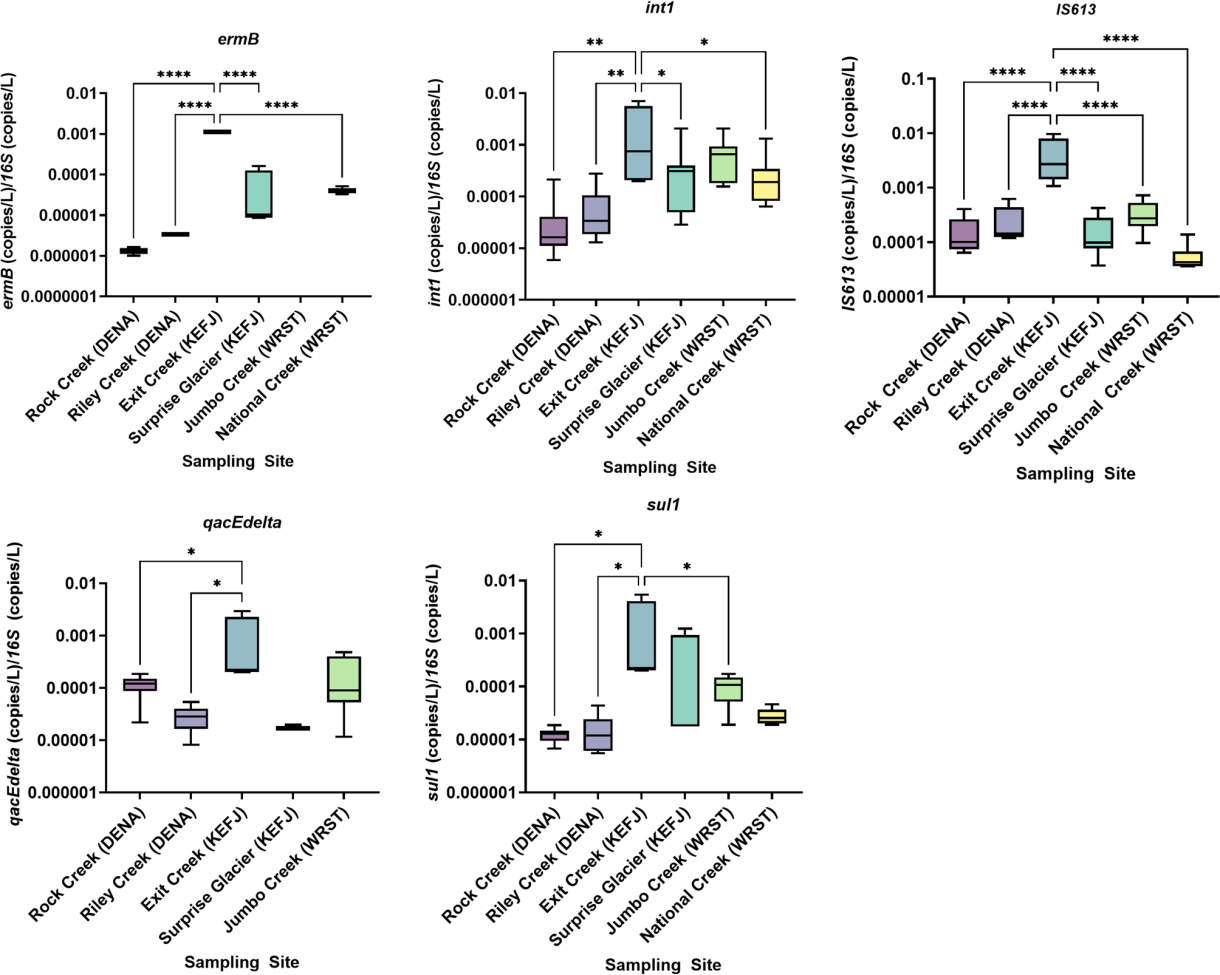
Relative abundance, ratios of antibiotic resistance gene concentrations (ARGs) to 16 S rRNA concentrations, for five ARGs that were detected at each of three national parks. Sample sites followed by DENA, KEFJ, and WRST were located in Denali National Park, Kenai Fjords National Park, and Wrangell St. Elias National Park respectively. Bars were divided at the median ARG/16S ratio and the hinges are at the 25th and 75th percentiles. Whiskers extend to the observed minimum and maximum ARG/16S ratios at each site. Site comparisons denoted by one, two, and four asterisks represent statistical significance at an alpha of 0.05, 0.01, and < 0.0001 respectively using a one-way ANOVA with Tukey’s correction for multiple comparisons. Site comparisons with no asterisk were not statistically significant at an alpha equal to 0.05.

**Fig. 6:**
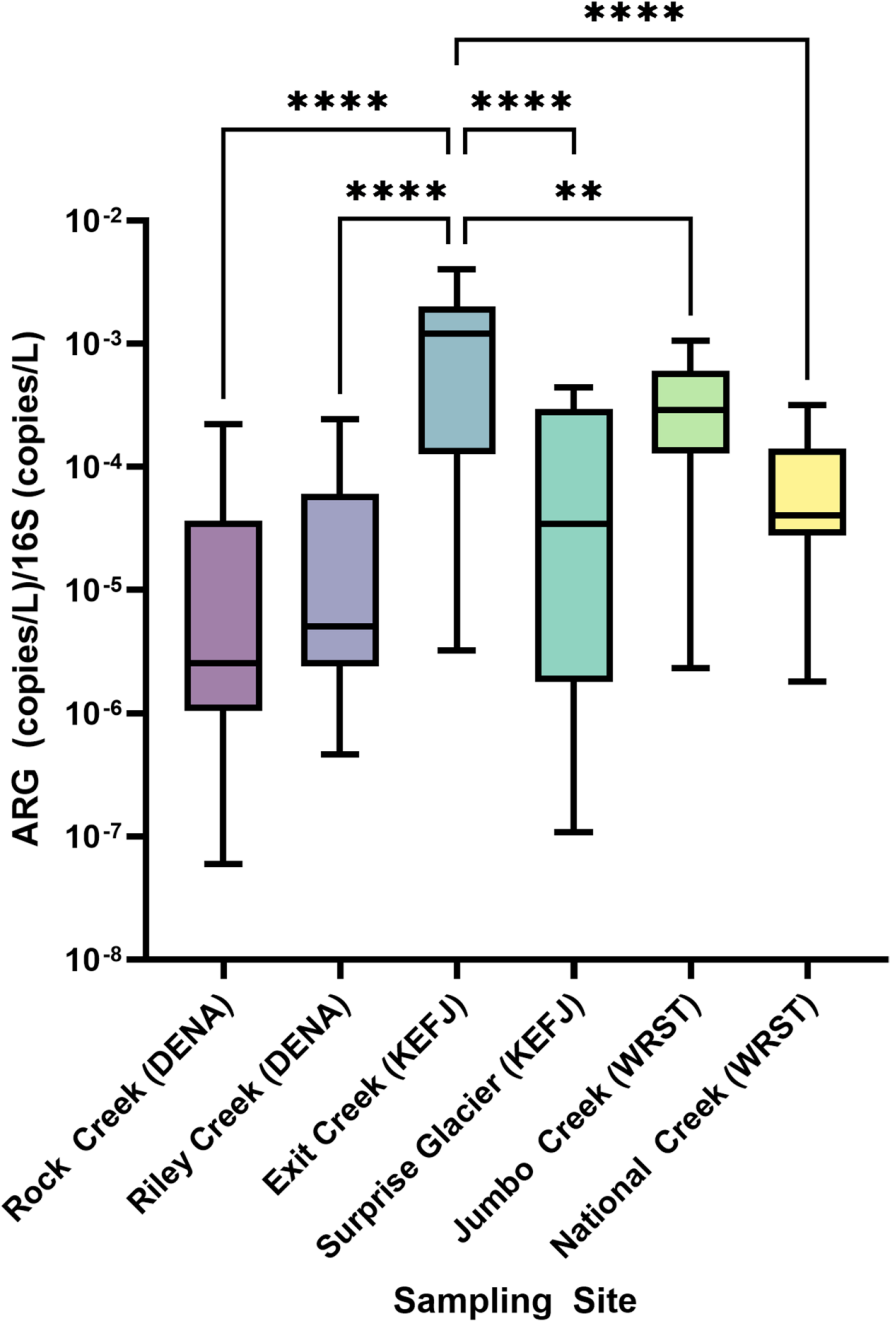
Relative gene target abundances at each sampling site. Sample names followed by DENA, KEFJ, and WRST were located at Denali National Park, Kenai Fjords National Park, and Wrangell St. Elias National Park respectively. Boxes are divided at the median and hinges are drawn at the 25th and 75th percentiles. Whiskers extend to the minimum and maximum observed ARG/16S rRNA ratios. Statistical significance in a one-way ANOVA test with the Tukey correction for multiple comparisons is denoted by two and four asterisks for *p* = 0.01 and *p* < 0.0001 respectively. All other comparisons were not statistically significant (*p* > 0.05).

We quantified the relative abundance of ARGs in each sample by comparing ratios of concentrations of ARGs detected with high confidence at all national park sites to 16 S concentrations. Concentrations of detected 16 S rRNA copies were compared among sites to determine if bacterial taxa distribution or bacterial load might skew proportional ARG outcomes (Supplementary Fig. 7). 16 S rRNA copies were higher at the Rock Creek site in Denali National Park than all sites at other parks, but not significantly different than the other Denali National Park site, Riley Creek. All other 16 S rRNA concentrations were not significantly different. The highest proportions of ARGs were consistently measured from Exit Creek at Kenai Fjords National Park (Fig. 5). A one-way ANOVA was used to determine if the mean ARG/16S ratios were different between any of the sampling sites. Significant differences in all high confidence detection ARG/16S ratios were between Exit Creek and every other sampling site (Rock Creek *p* < 0.0001, Riley Creek *p* < 0.0001, Surprise Glacier *p* < 0.0001, Jumbo Creek *p* = 0.0022, and National Creek *p* < 0.0001) (Fig. 6). All other site-to-site comparisons were not statistically significant (Supplementary Table 5). The relative abundance of the five ARGs detected with high confidence in each park (Fig. 5) were also compared site-to-site. For each of the ARGs, the relative abundance detected at Exit Creek was significantly higher than at least two other sampling sites. No other significant differences were detected.

**Fig. 7:**
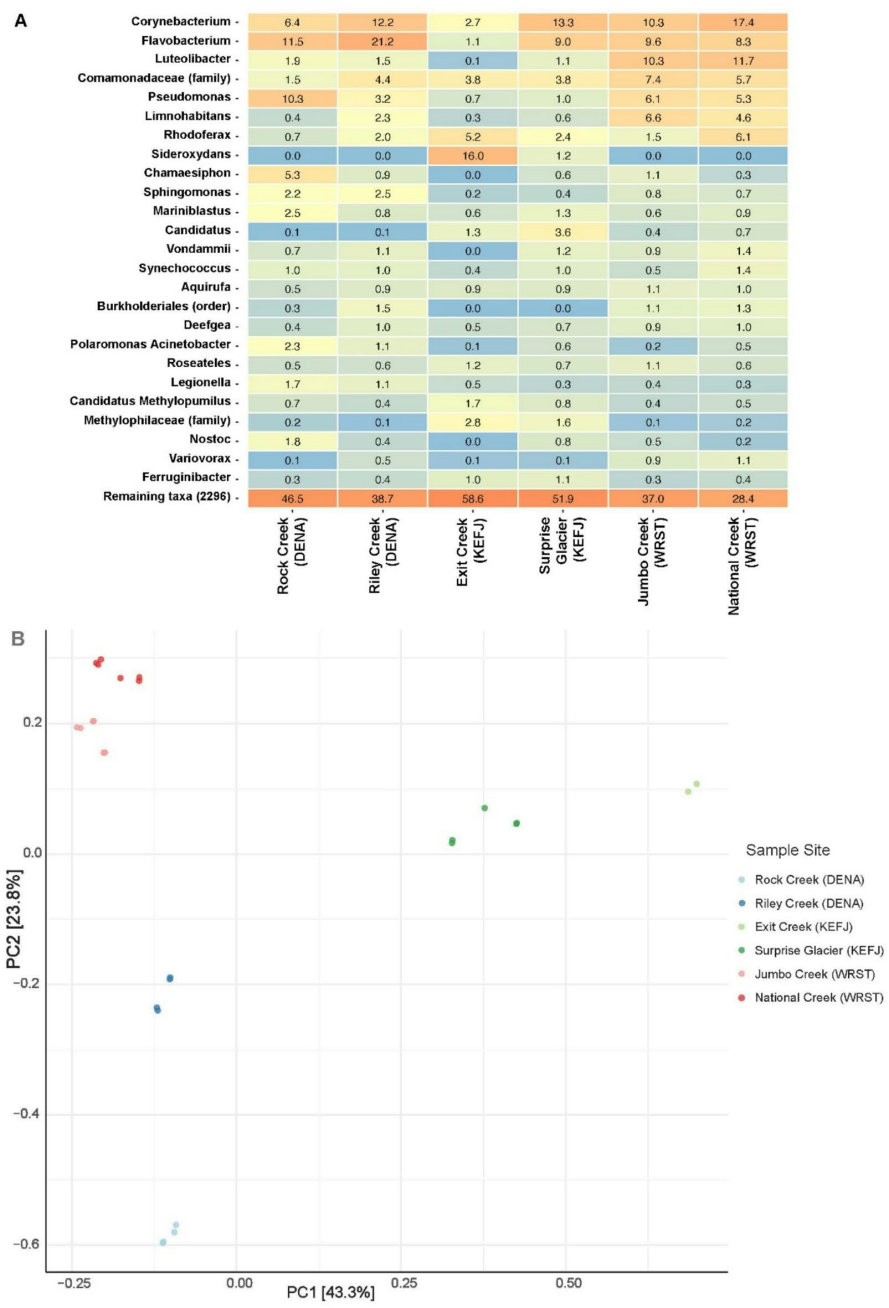
Relative read abundance of top 25 bacterial genera detected in surface waters from six sites in Denali National Park (DENA), Kenai Fjords National Park (KEFJ), and Wrangell-St. Elias National Park (WRST). Warmer cell colors indicate larger percent read abundance, while cooler colors indicate lower percent read abundance. (**A**) Principal components analysis of 16 S rRNA diversity in the same sites (**B**).

Bacterial taxonomic diversity was assessed using 16 S rRNA metagenomics. The most commonly detected genera tended to be primarily non-human pathogens (e.g., *Flavobacterium*,* Rhodoferax*, and *Limnohabitans*), however a few genera containing human pathogens were detected in the 25 most commonly detected genera (e.g., *Pseudomonas* and *Legionella*) (Fig. 7). Other pathogenic genera including *Staphylococcus*, *Enterococcus*, *Escherichia*, *Klebsiella*, and *Campylobacter* were detected in low relative abundance^59^. A PCA of these results indicates clustering of sites within the same national park, particularly in Wrangell-St. Elias National Park (Fig. 7).

## Discussion

The developed qPCR assay exhibited high sensitivity and specificity with near perfect agreement to whole genome sequencing of bacterial isolates for the detection of targeted ARGs. Compared to a commercially available qPCR AMR assay, our assay had a higher sensitivity and agreement with WGS and an equivocal specificity^28^. The observed incongruencies illustrate the potential for decreased specificity with SYBR based qPCR assays. Converting to a probe-based assay could increase the specificity and ease the transition to use on a digital PCR platform but may also increase the cost per assay and limit the number of gene variants amplified. Digital PCR functionality could be advantageous however, given the precise absolute quantitation and increased target sensitivity of these platforms. Furthermore, more investigation into melt curve analysis of variant gene targets with the current SYBR based format could increase the number of variants detected by a single primer set^60^.

Using ultrafiltration and the designed qPCR assay, ARGs were detected with high confidence and presumptively at three concentrations from spiked samples between 1 and 50 L. Unsurprisingly, larger volume samples yielded more high confidence detections though this trend was less evident when also considering presumptive positives. Consistent trends in detection among spiked bacterial concentration were not apparent. Given that bacterial growth was not inhibited as a part of the spikes and several of the bacteria used in this study have rapid doubling times, inconsistencies in spike concentration estimates may have occurred due to the inherent time between when spike-ins occurred, and filtration was completed, a limitation of this study design. More precise bacterial abundances could be achieved with additional quantitation experiments. Detection probabilities in the spikes decreased markedly from the comparison to WGS using bacterial isolates. These reduced detection probabilities are indicative of lower concentrations of genetic material and loss of target that occurs in the filtration, backflush, and concentration process. Of note, the ARG target *ermD* was not detected in any spike samples. The *ermD* gene was carried by the species *Geobacillus stearothermophilus* and *Pseudomonas putida*, and yielded cycle threshold values higher than most other detected genes from concentrated cultures, including other gene targets held by the same bacterium^61^. Despite similar cycle threshold values of gene targets carried by other bacteria, inefficient DNA extraction may be a contributing factor. Fewer copies carried per genome resulting in a lower copy concentration spiked in than other genes, including 16 S which was used to estimate spike concentrations, may have also led to the non-detection of the *ermD* target.

As part of the spikes, relative quantities of ARG targets/16S rRNA ratios were assessed and were generally found to be in the expected range. However, measured ratios higher than the expected for *bla*_*TEM*_ and the false positive detection of this target in the WGS comparison study suggest that some detections of *bla*_*TEM*_ with this method may be spurious. Therefore, careful scrutinization of detections and relative abundances may be prudent for applications in which this target is a high priority. Further validation of this technique may also be warranted for this and other ARG targets for specific projects and applications. Such efforts might include determination of limit of detection for the ultrafiltration process for differing source water types (wetlands, streams, marine, effluents, etc.) and optimizing the backflush procedure to maximize target recovery.

ARGs were detected and quantified at six sites from three national parks in Alaska using dead-end hollow-fiber ultrafiltration of 20-L and 50-L surface water samples. The number and proportion of high confidence detections increased with 50-L volume samples, consistent with results of our spikes. Replicability of high-confidence detections between filter replicates was higher in 50-L samples than 20-L samples, and standard deviations of relative abundances between replicates were lower in 50-L samples than 20-L samples, providing further evidence that data resolution and precision likely improve with increasing sample volumes. For other low-biomass water sample types, such as drinking water, or when microbial targets are suspected to be rare, large volume samples up to 100 L have previously been recommended^62–64^. Human health risks posed by ARGs in recreational and environmental waters are still largely unknown and likely vary significantly based on the gene detected and its abundance^6,65,66^. Despite these unquantified risks, the detection of some targets in environmental water, like *bla*_*KPC*_, may have public health implications. To best assess for the presence of presumably rare, but important targets such as these, large volume samples (e.g., 50–100 L) could be considered. A limitation of this approach is that PCR inhibition was not evaluated but may have affected the detection of rare targets. Additionally, uneven bacterial taxonomic distributions among samples could artificially skew ARG/16S rRNA proportional results if extreme differences occurred, further highlighting the need for integration of standardized methods.

Interestingly, *mexF*, an ARG consistently found in samples from both sites at Wrangell-St. Elias National Park, has previously been associated with heavy metal resistance, including copper^67,68^, which was heavily mined in the area where sampling occurred. A tetracycline resistance gene known for being an indicator of fecal pollution, *tetD*, was detected with high confidence in both sites from Denali National Park and Kenai Fjords National Park but never in Wrangell-St. Elias National Park. These findings could be suggestive of different drivers of AMR at the park sites sampled. Surprisingly, the number of unique ARGs detected at the more remote site in Kenai Fjords National Park, the creek below Surprise Glacier, equaled or outnumbered (depending on whether sample volume was controlled for) its presumably higher human impact counterpart, Exit Creek, despite the site’s limited accessibility. ARGs have been detected in remote and deep seawater samples including the Antarctic and South China Sea and linked to anthropogenic activity or pollution^69–71^. This leads us to hypothesize that seawater may be influencing the occurrence of ARGs in this tidally influenced stream.

In this study, ARGs were found in surface water samples from national parks in Alaska at lower relative abundances compared to those measured in human impacted environments including wastewater and urban areas^18^ but similar to those from Rocky Mountain National Park in Colorado^72^. Further investigation of ARG abundances in surface waters on public lands throughout the United States and beyond may help to better elucidate patterns of occurrence of ARGs in surface waters at national and continental scales. The results of this study further demonstrate that qPCR detection of ARGs can indicate of the presence of AMR in surface waters and provide preliminary inference to the type of anthropogenic influences that may be contributing to ARG presence and abundance^7,18,73^. Additionally, assessment of ARGs and MSTs in national parks may offer insight into microbial contamination into public lands from visitation^22,23,27^.

Congruence among phenotypic and genotypic analytical pipelines was not particularly high in this study. These results suggest ARGs identified through qPCR may be predominantly found within bacteria of lower relevance to public health, which is also supported by our 16 S rRNA metagenomic results. The discrepancy between phenotypic and genomic results could be due to a variety of effects including viable but not culturable bacteria, dead bacteria, non-target bacteria, exogenous DNA possessing the ARGs detected with qPCR, or false positive qPCR detections. Instances of detection of a clinically significant ARG in qPCR but not in bacteria isolation, as with *bla*_*KPC*_, *bla*_*OXA*_, and *bla*_*CTX−M*_ in this study, may warrant further investigation through amplicon sequencing in subsequent studies to verify the detection and elucidate which variant of the ARG is present. This finding demonstrates an important limitation of qPCR for questions of clinical significance from environmental samples. Any clinical inference is incomplete.

Here we have described the validation and utility of a protocol for the assessment of AMR in surface waters. The tested qPCR assay displays high sensitivity, specificity, and agreement with whole genome sequencing, and is applicable to large and small volume ultrafiltered water samples. The combination of large volume ultrafiltered surface water samples from public lands analyzed through qPCR analysis appears to hold promise for elucidating trends in occurrence and abundance of AMR. Further optimization and application of these methods in a standardized manner may represent an important step towards harmonization of environmental research and monitoring for AMR within the United States and globally.

## Supplementary Information

Below is the link to the electronic supplementary material.

Supplementary Material 1

## Data Availability

Quantitative PCR data presented in this paper are publicly available at [https://doi.org/10.5066/P14G9MJW]^60^ . The sequencing data generated during the current study are available in NCBI (BioProject ID: PRJNA1214890).

## References

[1] Franklin, A. M. et al. A one health approach for monitoring antimicrobial resistance: developing a National freshwater pilot effort. *Front. Water* **6** (2024). 10.3389/frwa.2024.1359109 PMC11157689 38855419

[2] Larsson, D. G. J. & Flach, C-F. Antibiotic resistance in the environment. *Nat. Rev. Microbiol.* **20**, 257–269 (2022). 34737424 10.1038/s41579-021-00649-x PMC8567979

[3] Li, L-G., Huang, Q., Yin, X. & Zhang, T. Source tracking of antibiotic resistance genes in the environment — Challenges, progress, and prospects. *Water Res.* **185**, 116127 (2020). 33086465 10.1016/j.watres.2020.116127

[4] Liguori, K. et al. Antimicrobial resistance monitoring of water environments: A framework for standardized methods and quality control. *Environ. Sci. Technol.* **56**, 9149–9160 (2022). 35732277 10.1021/acs.est.1c08918 PMC9261269

[5] Milligan, E. G. et al. A systematic review of Culture-Based methods for monitoring Antibiotic-Resistant Acinetobacter, Aeromonas, and Pseudomonas as environmentally relevant pathogens in wastewater and surface water. *Curr. Environ. Health Rep.* **10**, 154–171 (2023). 36821031 10.1007/s40572-023-00393-9 PMC10299953

[6] Nappier, S. P., Liguori, K., Ichida, A. M., Stewart, J. R. & Jones, K. R. Antibiotic resistance in recreational waters: state of the science. *Int. J. Environ. Res. Public. Health* **17** (2020). 10.3390/ijerph17218034 PMC7663426 33142796

[7] Chen, Z. et al. Unraveling the influence of human fecal pollution on antibiotic resistance gene levels in different receiving water bodies using crassphage indicator gene. *J. Hazard. Mater.* **442**, 130005 (2023). 36179618 10.1016/j.jhazmat.2022.130005

[8] Drane, K., Sheehan, M., Whelan, A., Ariel, E. & Kinobe, R. The role of wastewater treatment plants in dissemination of antibiotic resistance: Source, Measurement, removal and risk assessment. *Antibiotics* **13**, 668 (2024). 39061350 10.3390/antibiotics13070668 PMC11274174

[9] Kim, J. J. et al. Persistence of antibiotic resistance from animal agricultural effluents to surface water revealed by genome-centric metagenomics. *J. Hazard. Mater.* **457**, 131761 (2023). 37290355 10.1016/j.jhazmat.2023.131761

[10] Wang, Z., Cai, M., Du, P. & Li, X. Wastewater surveillance for antibiotics and resistance genes in a river catchment: Spatiotemporal variations and the main drivers. *Water Res.* **251**, 121090 (2024). 38219685 10.1016/j.watres.2023.121090

[11] Burch, T. R. et al. Microbial source tracking and land use associations for antibiotic resistance genes in private wells influenced by human and livestock fecal sources. *J. Environ. Qual.* **52**, 270–286 (2023). 36479898 10.1002/jeq2.20443

[12] Kocurek, B. et al. Metagenomic survey of antimicrobial resistance (AMR) in Maryland surface waters differentiated by high and low human impact. *Microbiol. Resource Announcements*. **13**, e00477–e00423 (2023). 10.1128/MRA.00477-23 PMC10793305 38032210

[13] Nolan, T. M. et al. Land use as a critical determinant of faecal and antimicrobial resistance gene pollution in riverine systems. *Sci. Total Environ.* **871**, 162052 (2023). 36758688 10.1016/j.scitotenv.2023.162052

[14] Gupta, S., Graham, D. W., Sreekrishnan, T. R. & Ahammad, S. Z. Heavy metal and antibiotic resistance in four Indian and UK rivers with different levels and types of water pollution. *Sci. Total Environ.* **857**, 159059 (2023). 36174689 10.1016/j.scitotenv.2022.159059

[15] Liu, S., Wang, P., Wang, X. & Chen, J. Ecological insights into the elevational biogeography of antibiotic resistance genes in a pristine river: metagenomic analysis along the Yarlung Tsangpo river on the Tibetan plateau. *Environ. Pollut.* **286**, 117101 (2021). 33971467 10.1016/j.envpol.2021.117101

[16] Reichert, G. et al. Determination of antibiotic resistance genes in a WWTP-impacted river in surface water, sediment, and biofilm: influence of seasonality and water quality. *Sci. Total Environ.* **768**, 144526 (2021). 33450684 10.1016/j.scitotenv.2020.144526

[17] Vadde, K. K., McCarthy, A. J., Rong, R. & Sekar, R. Quantification of microbial source tracking and pathogenic bacterial markers in water and sediments of Tiaoxi river (Taihu watershed). *Front. Microbiol.* **10 **(2019). 10.3389/fmicb.2019.00699 PMC6492492 31105648

[18] Abramova, A., Berendonk, T. U. & Bengtsson-Palme, J. A global baseline for qPCR-determined antimicrobial resistance gene prevalence across environments. *Environ. Int.* **178**, 108084 (2023). 37421899 10.1016/j.envint.2023.108084

[19] D’Costa, V. M. et al. Antibiotic resistance is ancient. *Nature* **477**, 457–461 (2011). 21881561 10.1038/nature10388

[20] Rothrock, M. J. Jr et al. How should we be determining background and baseline antibiotic resistance levels in agroecosystem research? *J. Environ. Qual.* **45**, 420–431 (2016). 27065388 10.2134/jeq2015.06.0327

[21] Baron, J. S. et al. Marmots do not drink coffee: human urine contributions to the nitrogen budget of a popular National park destination. *Ecosphere* **14**, e4504 (2023).

[22] Clow, D. W. et al. Effects of stock use and backpackers on water quality in wilderness in sequoia and kings Canyon National Parks, USA. *Environ. Manage.* **52**, 1400–1414 (2013). 24071726 10.1007/s00267-013-0166-x

[23] Forrester, H., Clow, D., Roche, J., Heyvaert, A. & Battaglin, W. Effects of backpacker Use, pack stock trail Use, and pack stock grazing on Water-Quality Indicators, including Nutrients, E. coli, Hormones, and Pharmaceuticals, in Yosemite National park. *USA Environ. Manage.* **60**, 526–543 (2017). 28597051 10.1007/s00267-017-0899-z

[24] Goodwin, K., Loso, M. G. & Braun, M. Glacial transport of human waste and survival of fecal bacteria on Mt. McKinley’s Kahiltna Glacier, Denali National Park, Alaska. *Arct. Antarct. Alp. Res.* **44**, 432–445 (2012).

[25] Griffin, D. W., Gray, M. A., Lyles, M. B. & Northup, D. E. The transport of nonindigenous microorganisms into caves by human visitation: A case study at Carlsbad caverns National park. *Geomicrobiol J.* **31**, 175–185 (2014).

[26] Moldovan, O. T. et al. Management of water bodies in show caves – A microbial approach. *Tour. Manag.* **78**, 104037 (2020).

[27] Scott, L. C. et al. Use of microbial fecal indicator monitoring and fecal source tracking as a park management tool in Rocky mountain National park. *Environ. Challenges*. **8**, 100583 (2022).

[28] Ahlstrom, C. A., Scott, L. C., Woksepp, H., Bonnedahl, J. & Ramey, A. M. Environmental antimicrobial resistance gene detection from wild bird habitats using two methods: A commercially available culture-independent qPCR assay and culture of indicator bacteria followed by whole-genome sequencing. *J. Global Antimicrob. Resist.* **33**, 186–193 (2023). 10.1016/j.jgar.2023.03.009 36972752

[29] Rochegüe, T. et al. An inventory of 44 qPCR assays using hydrolysis probes operating with a unique amplification condition for the detection and quantification of antibiotic resistance genes. *Diagn. Microbiol. Infect. Dis.* **100**, 115328 (2021). 33819858 10.1016/j.diagmicrobio.2021.115328

[30] Ng, L. K., Martin, I., Alfa, M. & Mulvey, M. Multiplex PCR for the detection of Tetracycline resistant genes. *Mol. Cell Probes*. **15**, 209–215 (2001). 11513555 10.1006/mcpr.2001.0363

[31] Poirel, L., Walsh, T. R., Cuvillier, V. & Nordmann, P. Multiplex PCR for detection of acquired carbapenemase genes. *Diagn. Microbiol. Infect. Dis.* **70**, 119–123 (2011). 21398074 10.1016/j.diagmicrobio.2010.12.002

[32] Tolosi, R. et al. Rapid detection and quantification of plasmid-mediated colistin resistance genes (mcr‐1 to mcr‐5) by real‐time PCR in bacterial and environmental samples. *J. Appl. Microbiol.* **129**, 1523–1529 (2020). 32510809 10.1111/jam.14738

[33] Hozalski Raymond, M., Zhao, X., Kim, T. & LaPara Timothy, M. On-site filtration of large sample volumes improves the detection of opportunistic pathogens in drinking water distribution systems. *Appl. Environ. Microbiol.* **90**, e01658–e01623 (2024). 38236032 10.1128/aem.01658-23 PMC10880612

[34] Kahler, A. M. et al. Evaluation of an Ultrafiltration-Based procedure for simultaneous recovery of diverse microbes in source waters. *Water* **7**, 1202–1216 (2015). 26530003 10.3390/w7031202 PMC4627901

[35] Korajkic, A., Kelleher, J., Shanks, O. C., Herrmann, M. P. & McMinn, B. R. Effectiveness of two wastewater disinfection strategies for the removal of fecal indicator bacteria, bacteriophage, and enteric viral pathogens concentrated using dead-end Hollow fiber ultrafiltration (D-HFUF). *Sci. Total Environ.* **831**, 154861 (2022). 35358531 10.1016/j.scitotenv.2022.154861 PMC9291237

[36] Kraft, A. L. et al. A comparison of methods to detect low levels of Salmonella enterica in surface waters to support antimicrobial resistance surveillance efforts performed in multiple laboratories. *Sci. Total Environ.* **905**, 167189 (2023). 37748604 10.1016/j.scitotenv.2023.167189

[37] Li, X., Harwood Valerie, J., Nayak, B. & Weidhaas Jennifer, L. Ultrafiltration and microarray for detection of microbial source tracking marker and pathogen genes in riverine and marine systems. *Appl. Environ. Microbiol.* **82**, 1625–1635 (2016). 26729716 10.1128/AEM.02583-15 PMC4771337

[38] Liu, P. et al. Hollow-fiber ultrafiltration for simultaneous recovery of viruses, bacteria and parasites from reclaimed water. *J. Microbiol. Methods*. **88**, 155–161 (2012). 22108496 10.1016/j.mimet.2011.11.007

[39] Liu, P. et al. Development of Moore swab and ultrafiltration concentration and detection methods for *Salmonella typhi* and *Salmonella paratyphi* A in wastewater and application in Kolkata, India and Dhaka, Bangladesh. *Front. Microbiol.* **12** (2021). 10.3389/fmicb.2021.684094 PMC8320291 34335510

[40] Pascual-Benito, M. et al. Assessment of dead-end ultrafiltration for the detection and quantification of microbial indicators and pathogens in the drinking water treatment processes. *Int. J. Hyg. Environ Health*. **230**, 113628 (2020). 33038613 10.1016/j.ijheh.2020.113628

[41] Robla, J. et al. Determination of the efficiency of filtration of cultures from microalgae and bacteria using Hollow fiber filters. *Environ. Science: Water Res. Technol.* **7**, 1230–1239 (2021).

[42] Smith Carmela, M. & Hill Vincent, R. Dead-End Hollow-Fiber ultrafiltration for recovery of diverse microbes from water. *Appl. Environ. Microbiol.* **75**, 5284–5289 (2009). 19561183 10.1128/AEM.00456-09 PMC2725477

[43] Polaczyk, A. L. et al. Ultrafiltration-based techniques for rapid and simultaneous concentration of multiple microbe classes from 100-L tap water samples. *J. Microbiol. Methods*. **73**, 92–99 (2008). 18395278 10.1016/j.mimet.2008.02.014

[44] Gallardo, V. J., Morris, B. J. & Rhodes, E. R. The use of Hollow fiber Dialysis filters operated in axial flow mode for recovery of microorganisms in large volume water samples with high loadings of particulate matter. *J. Microbiol. Methods*. **160**, 143–153 (2019). 30974128 10.1016/j.mimet.2019.04.005 PMC6528822

[45] Mull, B. & Hill, V. R. Recovery of diverse microbes in high turbidity surface water samples using dead-end ultrafiltration. *J. Microbiol. Methods*. **91**, 429–433 (2012). 23064261 10.1016/j.mimet.2012.10.001 PMC4625380

[46] Nadkarni, M. A., Martin, F. E., Jacques, N. A. & Hunter, N. Determination of bacterial load by real-time PCR using a broad-range (universal) probe and primers set. *Microbiology* **148**, 257–266 (2002). 11782518 10.1099/00221287-148-1-257

[47] Sayers, E. W. et al. Database resources of the National center for biotechnology information. *Nucleic Acids Res.* **50**, D20–d6 (2022). 34850941 10.1093/nar/gkab1112 PMC8728269

[48] Alcock, B. P. et al. CARD 2023: expanded curation, support for machine learning, and resistome prediction at the comprehensive antibiotic resistance database. *Nucleic Acids Res.* **51**, D690–d9 (2023). 36263822 10.1093/nar/gkac920 PMC9825576

[49] Zankari, E. et al. Identification of acquired antimicrobial resistance genes. *J. Antimicrob. Chemother.* **67**, 2640–2644 (2012). 22782487 10.1093/jac/dks261 PMC3468078

[50] SAS Institute. *9.4. Cary* (SAS Institute Inc., 2013).

[51] Service, N. P. *National Park Service Visitor Use Statistics* (National Park Service, Department of the Interior, 2024). https://irma.nps.gov

[52] European Committee on Antimicrobial Susceptibility Testing. Breakpoint tables for interpretation of MICs and zone diameters. Version 14.0 (2024).

[53] Klindworth, A. et al. Evaluation of general 16S ribosomal RNA gene PCR primers for classical and next-generation sequencing-based diversity studies. *Nucleic Acids Res.* **41**, e1–e (2012). 22933715 10.1093/nar/gks808 PMC3592464

[54] Illumina. 16S Metagenomic Sequencing Library Preparation. Preparing 16S Ribosomal RNA Gene Amplicons for the Illumina MiSeq System (2016).

[55] Wood, D. E., Lu, J. & Langmead, B. Improved metagenomic analysis with kraken 2. *Genome Biol.* **20**, 257 (2019). 31779668 10.1186/s13059-019-1891-0 PMC6883579

[56] Pruitt, K. D., Tatusova, T., Brown, G. R. & Maglott, D. R. NCBI reference sequences (RefSeq): current status, new features and genome annotation policy. *Nucleic Acids Res.* **40**, D130–D135 (2012). Epub 20111124. 22121212 10.1093/nar/gkr1079 PMC3245008

[57] kraken-biom. Enabling interoperative format conversion for Kraken results 1.2 (2016).

[58] Andersen, K. S., Kirkegaard, R. H., Karst, S. M. & Albertsen, M. ampvis2: an R package to analyse and visualise 16S rRNA amplicon data. *BioRxiv* :299537 (2018).

[59] R Core Team. R:A language and environment for statistical computing. 4.2.0: R Foundation for Statistical Computing, Vienna, Austria. https://www.R-project.org/ (2022).

[60] Juul, S. et al. Validation and advantages of using novel RT-qPCR melting curve analysis assays for the identification of SARS-CoV-2 variants. *Sci. Rep.* **12**, 13069 (2022). 35906388 10.1038/s41598-022-17339-0 PMC9338320

[61] Scott, L. C. et al. Data for validation and application of standardized quantitative PCR assay for the assessment of antimicrobial resistance genes in surface water: U.S. Geological Survey data release. 10.5066/P14G9MJW (2025). 10.1038/s41598-026-35635-x PMC13057163 41771916

[62] Bridle, H., Jacobsson, K. & Schultz, A. C. Chapter 4 - Sample processing. In: (ed Bridle, H.) Waterborne Pathogens (Second Edition): Academic; 63–109. (2021).

[63] Forés, E. et al. Evaluation of a virus concentration method based on ultrafiltration and wet foam elution for studying viruses from large-volume water samples. *Sci. Total Environ.* **829**, 154431 (2022). 35278558 10.1016/j.scitotenv.2022.154431

[64] USEPA. 1060 Collection and Preservation of Samples. *Standard Methods for the Examination of Water and Wastewater. Standard Methods for the Examination of Water and Wastewater* (American Public Health Association, 2017).

[65] Amarasiri, M., Sano, D. & Suzuki, S. Understanding human health risks caused by antibiotic resistant bacteria (ARB) and antibiotic resistance genes (ARG) in water environments: current knowledge and questions to be answered. *Crit. Rev. Environ. Sci. Technol.* **50**, 2016–2059 (2020).

[66] Goh, S. G. et al. Assessing the additional health burden of antibiotic resistant Enterobacteriaceae in surface waters through an integrated QMRA and DALY approach. *J. Hazard. Mater.* **458**, 132058 (2023). 37459761 10.1016/j.jhazmat.2023.132058

[67] Chettri, U., Nongkhlaw, M. & Joshi, S. R. Molecular evidence for occurrence of heavy metal and antibiotic resistance genes among predominant metal tolerant Pseudomonas sp. and Serratia sp. Prevalent in the Teesta river. *Curr. Microbiol.* **80**, 226 (2023). 37227565 10.1007/s00284-023-03334-9

[68] Wang, P., Wu, D., You, X., Li, W. & Xie, B. Distribution of antibiotics, metals and antibiotic resistance genes during landfilling process in major municipal solid waste landfills. *Environ. Pollut.* **255**, 113222 (2019). 31563781 10.1016/j.envpol.2019.113222

[69] Blanco-Picazo, P. et al. Antibiotic resistance genes in phage particles from Antarctic and mediterranean seawater ecosystems. *Microorganisms* **8**, 1293 (2020). 32847015 10.3390/microorganisms8091293 PMC7565065

[70] Jang, J. et al. Abundance and diversity of antibiotic resistance genes and bacterial communities in the Western Pacific and Southern oceans. *Sci. Total Environ.* **822**, 153360 (2022). 35085628 10.1016/j.scitotenv.2022.153360

[71] Lu, J., Wu, J., Zhang, C., Wang, J. & He, X. Occurrence and possible sources of antibiotic resistance genes in seawater of the South China sea. *Front. Environ. Sci. Eng.* **18**, 108 (2024).

[72] Scott, L. C. et al. Assessing visitor use impact on antibiotic resistant bacteria and antibiotic resistance genes in soil and water environments of Rocky mountain National park. *Sci. Total Environ.* **785**, 147122 (2021). 33932658 10.1016/j.scitotenv.2021.147122

[73] Bengtsson-Palme, J., Kristiansson, E. & Larsson, D. G. J. Environmental factors influencing the development and spread of antibiotic resistance. *FEMS Microbiol. Rev.* **42** (2018). 10.1093/femsre/fux053 PMC5812547 29069382

